# An intensive multilocation temporal dataset of fungal and bacterial communities in the root and rhizosphere of *Brassica napus*

**DOI:** 10.1016/j.dib.2020.106143

**Published:** 2020-08-07

**Authors:** Navid Bazghaleh, Jennifer K. Bell, Steven D. Mamet, Zayda Morales Moreira, Zelalem M. Taye, Shanay Williams, Charlotte Norris, Tanner Dowhy, Melissa Arcand, Eric G. Lamb, Matthew Links, Steve Shirtliffe, Sally Vail, Steven D. Siciliano, Bobbi Helgason

**Affiliations:** a Department of Soil Science, University of Saskatchewan, 51 Campus Drive, S7N 5A8 Saskatoon Saskatchewan, Canada; b Department of Food and Bioproduct Sciences, University of Saskatchewan, 51 Campus Drive, S7N 5A8 Saskatoon Saskatchewan, Canada; c Department of Plant Sciences, University of Saskatchewan, 51 Campus Drive, S7N 5A8 Saskatoon Saskatchewan, Canada; d Department of Animal and Poultry Science, University of Saskatchewan, 51 Campus Drive, S7N 5A8 Saskatoon Saskatchewan, Canada; e Agriculture and Agri-food Canada, 107 Science Pl, S7N 5A8 Saskatoon Saskatchewan, Canada

**Keywords:** Canola, *Brassica napus* L., Bacterial and Fungal Microbiome, Rhizosphere, Root

## Abstract

The plant microbiome has been recently recognized as a plant phenotype to help in the food security of the future population. However, global plant microbiome datasets are insufficient to be used effectively for breeding this new generation of crop plants. We surveyed the diversity and temporal composition of bacterial and fungal communities in the root and rhizosphere of *Brassica napus*, the world's second largest oilseed crop, weekly in eight diverse lines at one site and every three weeks in sixteen lines, at three sites in 2016 and 2017 in the Canadian Prairies. We sequenced the bacterial 16S ribosomal RNA gene generating a total of 127.7 million reads and the fungal internal transcribed spacer (ITS) region generating 113.4 million reads. 14,944 unique fungal amplicon sequence variants (ASV) were detected, with an average of 43 ASVs per root and 105 ASVs per rhizosphere sample. We detected 10,882 unique bacterial ASVs with an average of 249 ASVs per sample. Temporal, site-to-site, and line-driven variability were key determinants of microbial community structure. This dataset is a valuable resource to systematically extract information on the belowground microbiome of diverse *B. napus* lines in different environments, at different times in the growing season, in order to adapt effective varieties for sustainable crop production systems.

## Specifications Table

**Table:** 

Subject	Agriculture, Crop production, Applied microbiology
Specific subject area	Diversity and temporal composition of bacterial and fungal communities in the root and rhizosphere of *Brassica napus*
Type of data	Figure
How data were acquired	DNA sequences: Illumina Miseq platform Data processing: QIIME2 platform v. 2019.1. Data analysis: R v. 3.6.1.
Data format	Raw and analyzed: (*.txt)
Parameters for data collection	Crop: sixteen diverse lines of *Brassica napus* Field sites: three sites in the Canadian prairies Materials: Root and Rhizosphere Sampling time: weekly at one site and every three weeks at three sites Years: 2016 and 2017
Description of data collection	Root and rhizosphere soil were collected and used for DNA library preparation based on amplicon sequencing of the 16s rRNA and Internal Transcribed Spacer (ITS).
Data source location	City/province (1): Llewellyn / Saskatchewan (52.1718° N, 106.5052° W) City/province (2): Melfort / Saskatchewan (52.8185° N, 104.6027° W) City/province (3): Scott / Saskatchewan (52.3574° N, 108.8400° W) Country: Canada
Data accessibility	Fungal data Repository name: Harvard Dataverse Data identification number: https://doi.org/10.7910/DVN/DW2IUT Direct URL to data: https://dataverse.harvard.edu/dataset.xhtml?persistentId=doi:10.7910/DVN/DW2IUT Raw sequences Repository name: NCBI SRA Data identification number: PRJNA575004 Accessions: SAMN13414364 - SAMN13415317; SAMN13416986 - SAMN13417833; SAMN13416203 - SAMN13416971 Bacterial data Repository name: Dryad Data Identification number: https://doi.org/10.5061/dryad.30t86d1 Direct URL to data: https://datadryad.org/stash/dataset//doi:10.5061/dryad.30t86d1 Raw sequences Repository name: NCBI SRA Data identification number: PRJNA575004 Accessions: SAMN12874189 – SAMN12875128; SAMN12898547 – SAMN12899542; SAMN12907026 – SAMN12907756

## Value of the Data

• This dataset characterizes the bacterial and fungal microbiomes in the root endosphere and rhizosphere of *B. napus* in the Canadian prairies.
• It can be used to systematically extract information on diversity and composition of the root and rhizosphere microbiome in diverse *B. napus* lines, in different environments, and at different times in the growing season.
• The data presented in this article are useful in various areas including microbial ecology, soil science, plant science, and in breeding programs as an alternative plant phenotype, in order to adapt effective varieties for sustainable crop production.

## 1 Data Description

This dataset provides information on the diversity and temporal composition of the bacterial and fungal microbiomes in the root endosphere and rhizosphere of *Brassica napus* L. in different environments in the Canadian prairies. Previous reports revealed that root microbiomes of *B. napus*, the world's second largest oilseed crop, was consistently different from those of other crop plants, and tended to shift in different cropping rotation systems [[1], [2], [3], [4]]. However, no evidence was provided on the controlling factors, including how plant genetics and environment may shape the diversity and composition of these microbiomes [5,6].

We surveyed the diversity and composition of the root and rhizosphere bacterial and fungal microbiomes of sixteen genetically diverse *B. napus* lines replicated three times. A total for four site years included sites at Llewelyn in 2016 and 2017 as well as Melfort and Scott in 2017 only. A temporally intensive survey was performed once per week for ten weeks in 2016 at Llewelyn to determine the degree of change in the bacterial and fungal microbiomes over the growing season. In 2017, we repeated this work at three time points with the same 16 lines grown in multiple locations (Llewelyn, Melfort and Scott). These locations had different soils and climatic factors that are representative of important canola producing regions in the Canadian prairies. In 2017, we also repeated the weekly sampling of a subset of eight lines at the Llewelyn field site (Table 1).

**Table 1:** Description of the *B. napus* lines used in this study.

*B. napus* Line	Description	Origin	Sampled at weeks 3, 6 and 9	Sampled weekly
NAM 0	Breeding Line	Canada	√	√
NAM 13	Cultivar	Germany	√	√
NAM 14	Cultivar	Sweden	√	
NAM 17	Breeding Line	Canada	√	√
NAM 23	Accession	North Korea	√	
NAM 30	Cultivar	European	√	
NAM 32	Accession	South Korea	√	√
NAM 37	Cultivar	Australia	√	√
NAM 43	Accession	Bangladesh	√	√
NAM 46	Accession	South Korea	√	
NAM 5	Accession	India	√	
NAM 72	Breeding Line	Canada	√	√
NAM 76	Cultivar	Canada	√	
NAM 79	Accession	Pakistan	√	
NAM 48	Breeding Line	Canada	√	
YN04-C1213	Breeding Line	Canada	√	√

**Fig. 1:**
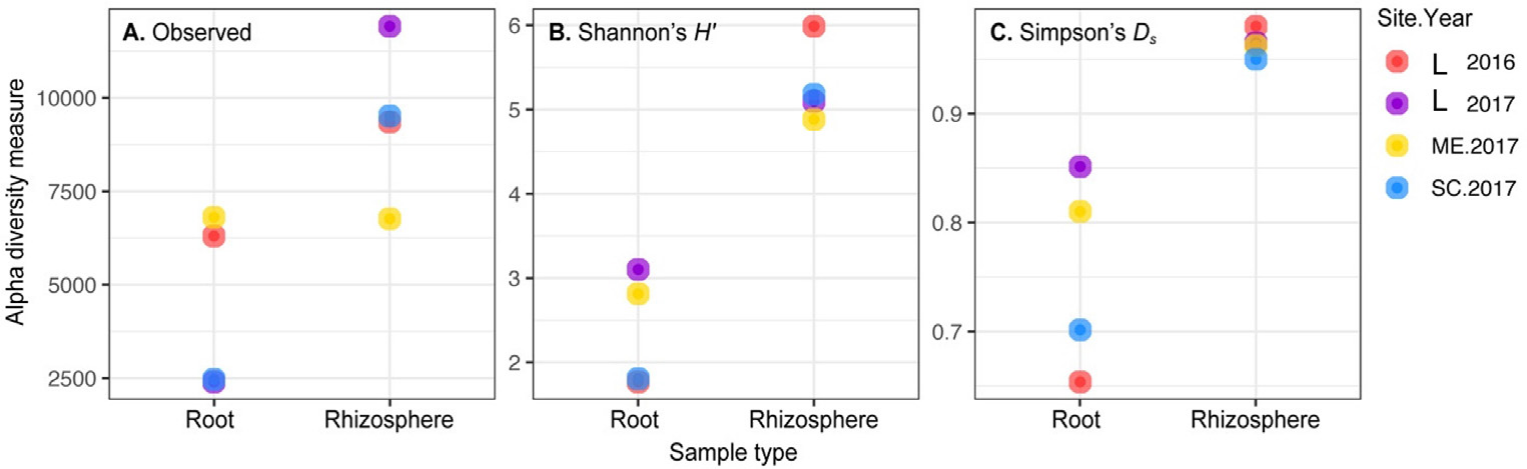
Bacterial alpha diversity analyses. (A) The number of unique taxa in each sample type. (B) Shannon diversity index (H′), which combines evenness and richness into a single measure. (C) Simpson's index (D_s_), which is the probability that two randomly sampled taxa are from two different groups. Alphanumeric codes for each represent the alpha diversity of each site in a given year. SK refers to Saskatoon, ME to Melfort and SC to Scott with the year sampled indicated.

**Fig. 2:**
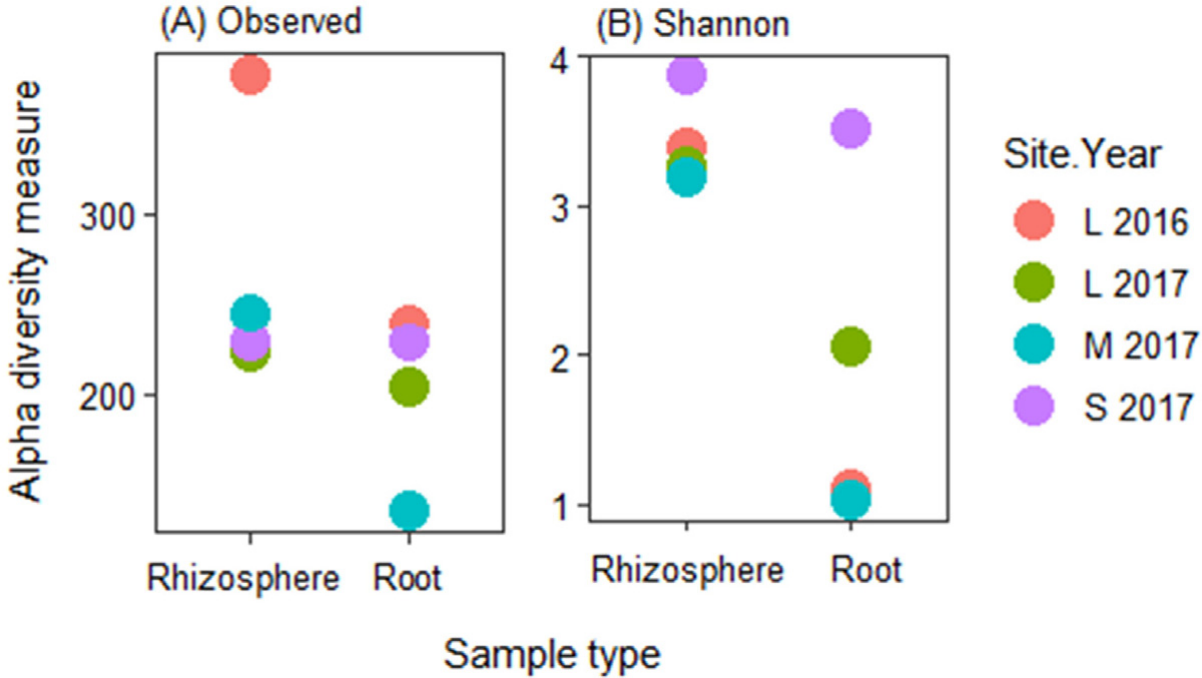
Fungal alpha diversity in each site in a given year. (A) Observed species, and (B) Shannon's *H* index, explaining the diversity of fungal taxa. L refers to Llewellyn, M to Melfort, and S to Scott in 2016 and 2017.

**Fig 3:**
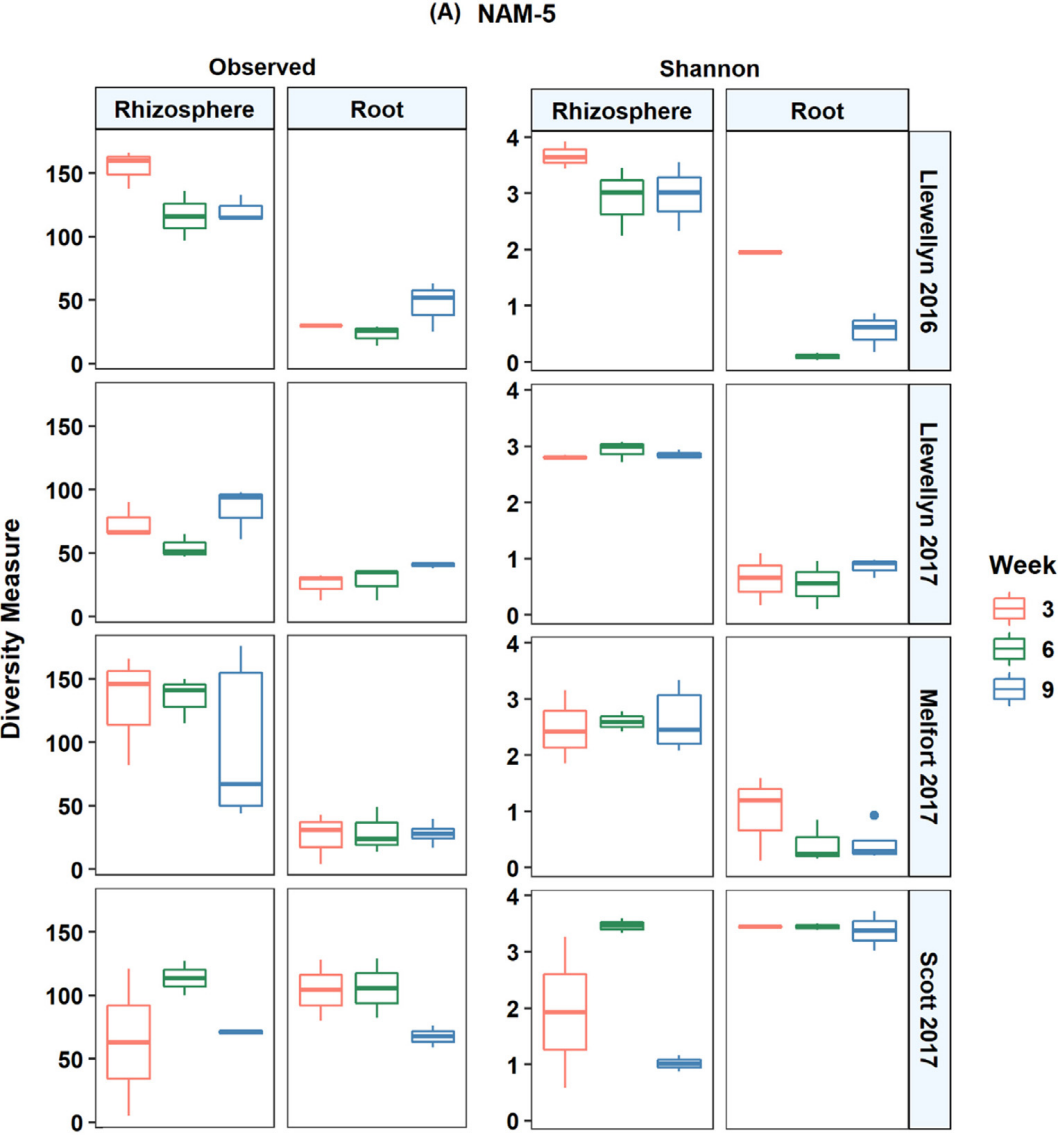

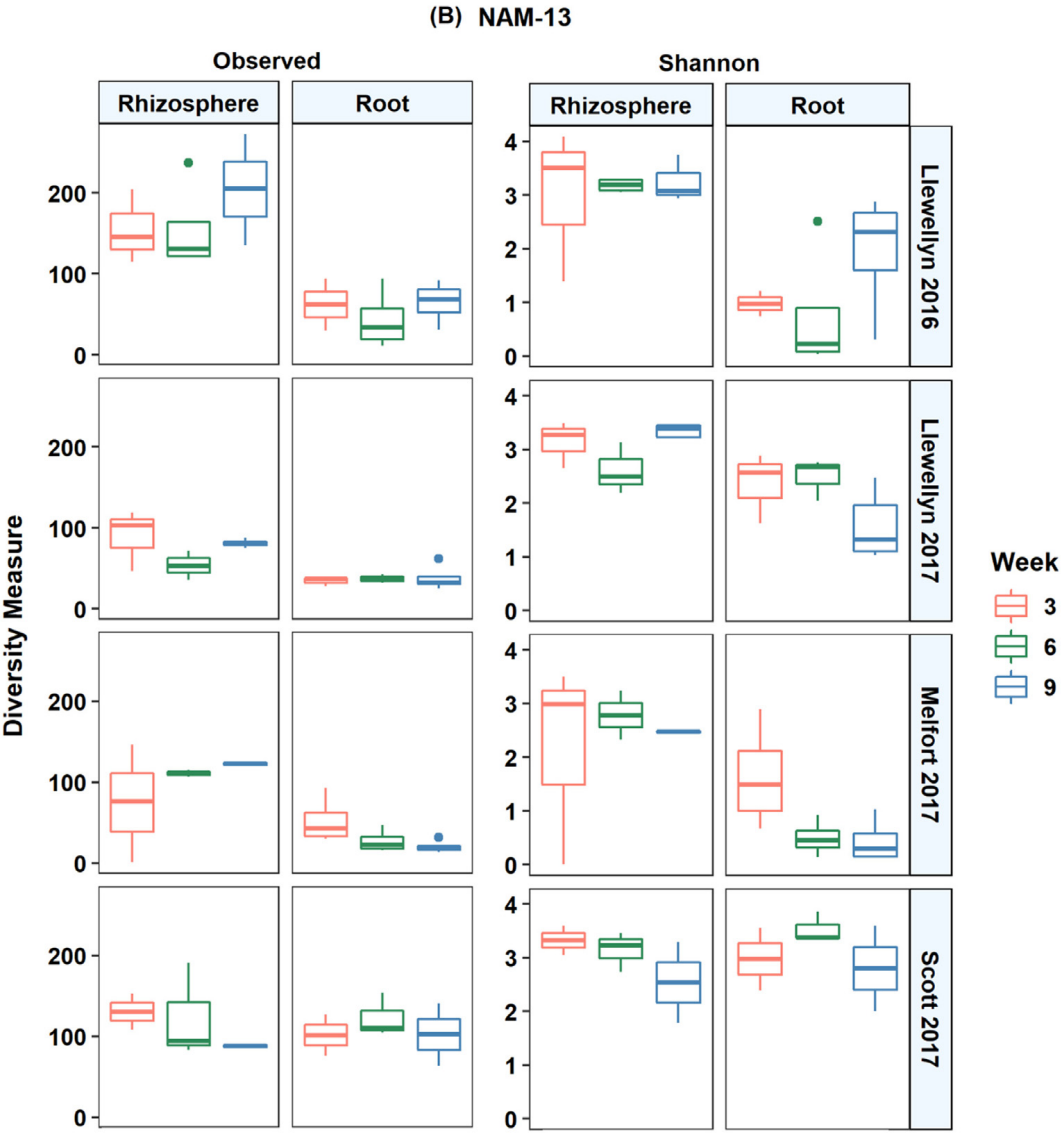
Fungal alpha diversity in two canola lines over growing seasons in each site in a given year. Observed taxa and Shannon's *H* index of diversity of fungal taxa in the roots and rhizosphere of two canola lines including NAM-5 (A) and NAM 13 (B) at weeks 3, 6, and 9 in 2016 and 2017.

**Fig. 4:**
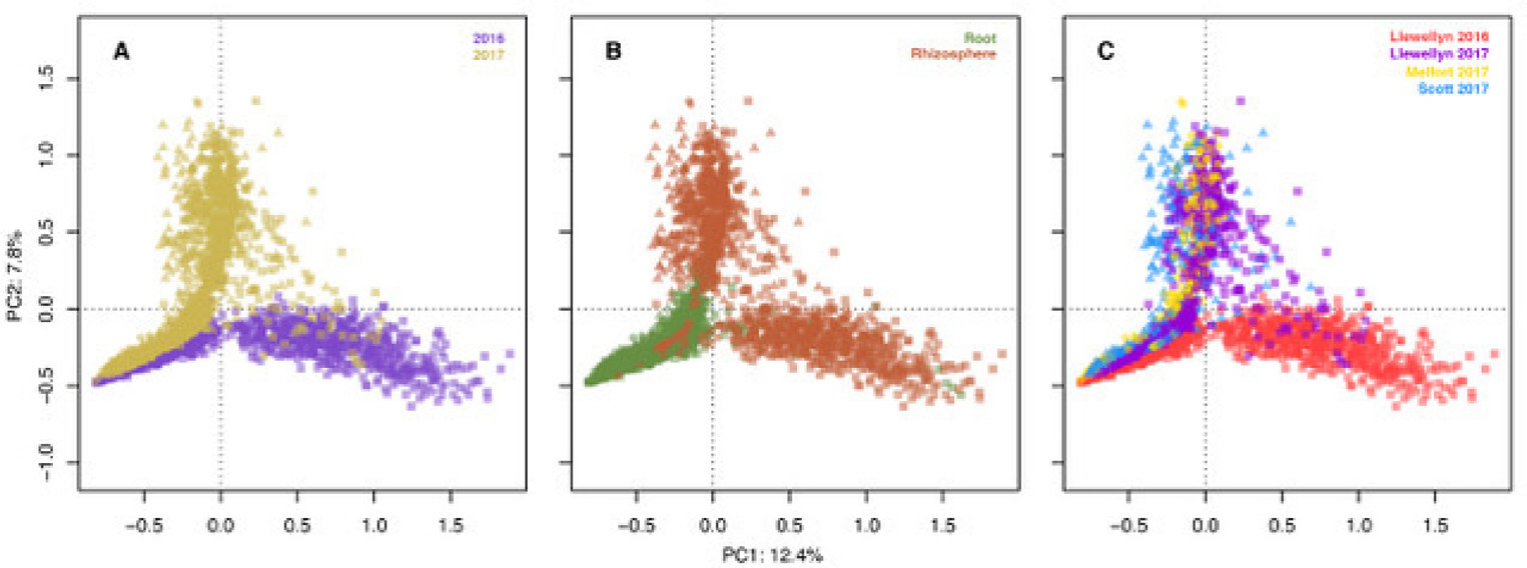
Principal components analysis. A total of 2594 samples were analyzed using 16S rRNA V3/V4 amplicon sequencing. (A) There were 1120 samples included from 2016 and 1474 from 2017. (B) Includes a total of 1302 root and 1292 rhizosphere samples. (C) The total samples per site in 2016 were: 1120 from Saskatoon. 2017: 739 from Saskatoon, 389 from Melfort, and 346 from Scott.

**Fig. 5:**
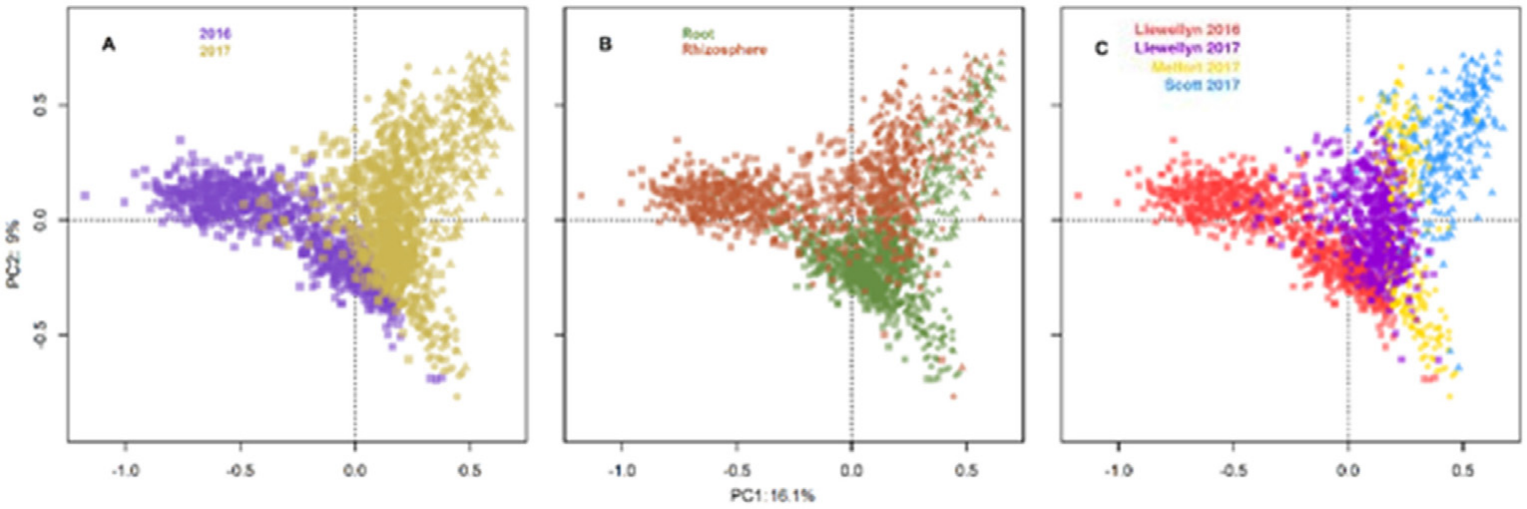
Principal components analysis of fungal taxa obtained using ITS amplicon sequencing in different years (A), sample types (B) and sites (C). Permutational Analysis of Variance (PERMANOVA) shows significant effects of year (*p*<0.001*)*, sample type (*p*<0.001*)*, and site by year (*p*<0.001*)* on fungal microbiome composition.

In total, 14,944 unique fungal and 10,882 unique bacterial amplicon sequence variants (ASV) were detected in all lines across the four site years based on the internal transcribed spacer region. There was an average of 43 fungal ASVs per root and 105 ASVs per rhizosphere sample. The bacterial and fungal microbiomes were more diverse in the rhizosphere samples compared to the root samples. In Llewelyn 2017, the total number of bacterial ASVs was the highest in rhizosphere samples whereas it was the lowest for the root samples (Fig. 1). The fungal microbiome was more diverse at Scott compared to Llewellyn and Melfort. In Llewellyn, the site with two years of data, the root fungal microbiome was more diverse in 2017 than 2016, while in contrast in the rhizosphere soil the microbiome had a higher richness in 2016 than 2017 (Fig. 2). Diversity of fungi varied in different *B. napus* lines and it shifted over growing seasons. An example of this is shown for lines NAM-5 and NAM-13 (Fig. 3). The composition of bacterial and fungal communities in the root and rhizosphere was shaped by *B. napus* line, location, and the year the experiment was conducted, likely dominated by year-to-year differences in environmental and edaphic conditions (Fig. 4, Fig. 5).

This dataset is a valuable resource that can be used to systematically extract information on the temporal dynamics of microbial communities in the root endosphere and rhizosphere soil of diverse *B. napus* lines in different soils and across years. The purpose of this report is to provide a publicly available microbiome dataset, its associated metadata and the context by which others explore how bacterial and fungal microbiomes relate to varieties better suited to sustainable crop production, and improve food security worldwide [7], [8].

## 2 Experimental Design, Materials, and Methods

### 2.1 Experimental design

Sixteen lines of *B. napus* L. were grown in Agriculture and Agri-Food Canada research farms in Saskatchewan in 2016 and 2017 (Table 1). In 2016, the lines were grown in a site at Llewellyn (52.1718° N, 106.5052° W). In 2017, the same lines were again grown at Llewellyn, as well as at Scott (52.3574° N, 108.8400° W) and Melfort (52.8185° N, 104.6027° W) (Fig. S1). Each line was grown in randomized blocks and replicated three times. Each plot had six rows and was 6.1 m long and 1.8 m wide. At Llewellyn in 2016, the plots were seeded on May 27 and received 220.1 mm of precipitation throughout the growing season (May to August) and had a mean temperature of 16.7 °C. In 2017, the plots were seeded on May 28-29 at Llewellyn and received 127.9 mm of precipitation throughout the growing season with a mean air temperature of 16.4 °C. The plots located in Llewellyn in 2017 were adjacent to the plots from 2016, but were not planted in the same exact physical location. At Melfort, the plots were seeded on May 19, 2017 and received 126.9 mm of precipitation throughout the growing season with a mean temperature of 15.5 °C. At the Scott site the plots were seeded on June 20, 2017 and received 178.7 mm precipitation with a mean temperature of 15.4 °C. These plots were initially seeded in May but the plants were lost due to a hailstorm and they were reseeded later in June.

In 2016, root and rhizosphere samples were collected weekly from June 14 to August 16 from the Llewellyn site for 10 consecutive weeks, starting 3 weeks after sowing. In addition, three duplicates were collected for randomly chosen samples at each sampling time throughout the growing season resulting in a total of 510 samples. In 2017, the same sixteen lines were sampled at all three sites 3, 6, and 9 weeks after sowing. In 2017, eight lines were also sampled weekly for 10 weeks from the site at Llewellyn, starting 3 weeks after sowing. Three canola plants were collected and combined to form a single composite sample from each plot. A total of 2,160 root and rhizosphere samples were collected and DNA was extracted from all samples. Roots and rhizosphere samples were collected to a 10-cm depth, and rhizosphere soil was determined as soils adhering to the roots.

### 2.2 Sample collection and processing

Three plants including roots were sampled from each plot using a sterilized trowel. Roots with adhering soil and bulk soil were placed in a sampling bag and stored in a cooler where they were kept on ice. The samples were transported to the lab and were stored at 4 °C until processing. The following day, the aboveground material was cut from the roots, oven dried at 60 °C, and weighed. The loosely adhering soil (soil not attached to the roots) was shaken off and collected and stored at -80 °C for further analysis. Plant roots and the tightly adhering soil were weighed and transferred to a flask containing 100 ml of sterile 0.05M NaCl buffer and shaken at 180 rpm for 15 minutes. The roots then were rinsed with deionized water, weighed, and a subsample of the root material was stored at -80 °C for DNA extraction. The buffer and soil mixture was centrifuged at 5000 rpm for 15 minutes at room temperature. The supernatant was stored at -20 °C for root exudate analysis. The pellet containing the rhizosphere soil was transferred to 1.5 ml tubes and stored at -80 °C for DNA extraction.

### 2.3 DNA extraction and amplification

DNA was extracted from 250 mg of rhizosphere soil using Qiagen PowerSoil extraction kit following manufacturer instructions. DNA was extracted from 50 mg root tissue using Qiagen PowerPlant extraction kit (Hilden, Germany) following manufacturer instructions. Extraction duplicates were included for quality control. After extraction, DNA quantity and quality were determined following the standard Qubit protocol (Thermo Fisher Scientific, Waltham Massachusetts). Prior to amplification, DNA from soil was standardized to 5 ng/μL, and DNA from roots was standardized to 1.5 ng/μL.

The V4 region of the 16S rRNA was amplified using the primer set 342F with Illumina adapters (5’ - TCGTCGGCAGCGTCAGATGTGTATAAGAGACAG CTA CGG GGG GCA GCA G - 3’) and the 806R (5’ - GTCTCGTGGGCTCGGAGATGTGTATAAGAGACAG GGA CTA CCG GGG TAT CT - 3’) [9]. In 2016, the PCR reaction mix (25 μl total) contained 2.5 μl DreamTaq Buffer (Thermo Fisher Scientific, Waltham Massachusetts), 2 μl MgCl_2_, 2.5 μl betaine, 2.5 μl dNTP mix (Invitrogen, Carlsbad, California), 1 μl of each primer, 0.25 μl DreamTaq (Thermo Fisher Scientific, Waltham Massachusetts), 13.25 μl nuclease free water, and 2 μl of the standardized template DNA. The PCR conditions were 95 °C for 5 minutes as an initial denaturization, followed by 95 °C for 30 seconds, 54 °C for 30 seconds, 72 °C for 30 seconds for 35 cycles, and a final elongation of 72 °C for 7 minutes. Negative controls and PCR duplicates were included. In 2017 the same reaction mix was used without MgCl_2_ and betaine. The same PCR conditions were followed.

The Internal Transcribed Spacer (ITS) region was amplified using the ITS1F_KYO1 (CTHGGTCATTTAGAGGAASTAA) / ITS2_KYO2 (TTYRCTRCGTTCTTCATC) primer set [10] and then sequenced using the Illumina MiSeq Platform. The PCR reaction mix (25 μL total) contained 12.5 μL Platinum Green Taq buffer (Invitrogen, Carlsbad, California), 1 μL of each of the forward and reverse primers (10 μM), 8.5 μL nuclease free water, and 2 μL of template DNA. The PCR conditions were 95 °C for 5 minutes as an initial denaturation, followed by 95 °C for 30 seconds, 51 °C for 45 seconds, 72 °C for 1 min for 35 cycles, and a final elongation of 72 °C for 7 minutes. PCR reactions with no DNA template were included for negative control. PCR reactions were replicated for a fraction of randomly selected DNA samples and used for PCR quality control.

### 2.4 Library preparation

For bacterial amplification, samples collected in 2016 were sent to Genome Quebec (Montreal, Quebec) after the initial PCR for barcoding, amplicon normalization, amplicon library QC, and sequencing on Illumina MiSeq platform (San Diego. California). Library preparation for 2017 samples was completed at the University of Saskatchewan. PCR product was purified to eliminate primers and impurities using 1:1 ratio of Nucleomag NGS clean-up and size select (D-mark Biosciences, Scarborough, Ontario). After purification, samples were indexed following the Illumina protocol, purified again to remove excess index primers, quantified and standardized to 4 nM, and pooled. Pooled libraries were then sequenced using the Illumina MiSeq platform. A total of 1,008 individual root samples and 1,008 rhizosphere soil samples, as well as 578 quality assurance/quality control samples were sequenced. Quality assurance/control samples included field soil and root duplicates, DNA extraction duplicates, library preparation duplicates and sequencing duplicates. On average, every third field experimental unit was duplicated in some fashion for quality assurance/control. The raw sequences were submitted to the sequence read archive (SRA) repository of the National Center for Biotechnology Information (BioProject PRJNA575004, accessions: SAMN12874189 – SAMN12875128; SAMN12898547 – SAMN12899542; SAMN12907026 – SAMN12907756).

For fungal amplification, the PCR products were confirmed by visualization in an agarose gel (1.2 %), purified using 1:1 ratio of Nucleomag NGS clean-up and size selected to remove primers and impurities according to manufacturer's instructions (D-mark Biosciences, Scarborough, Ontario). After purification, samples were barcoded using Nextera XT indexes, purified again to remove the impurities and indexing primers, quantified using Qubit 4 (Thermo Fisher Scientific, Waltham Massachusetts), standardized at 4 ng/μL, and pooled (384 samples). Selected PCR reactions were indexed separately and used for sequencing quality control.

Pooled libraries were then sequenced using the Illumina MiSeq platform using MiSeq Reagent Kit v2 (500-cycles). A total of 1,080 individual root samples and 1,080 rhizosphere soil samples, as well as 411 duplicates were sequenced. The raw sequences were submitted to the SRA repository of the NCBI (BioProject PRJNA575004, accessions: SAMN13414364 - SAMN13415317; SAMN13416986 - SAMN13417833; SAMN13416203 - SAMN13416971).

### 2.5 Bioinformatics

#### 2.5.1 Bacteria

A total of 127,698,986 reads were produced with an average of 23,940 reads per sample. Primers were removed using cutadapt v. 2.1 and then imported into QIIME2 v 2019.1 [11]. Sequences were then filtered based on quality using default parameters: maximum number of consecutive low-quality scores (*r*) = 3, minimum length that a sequence read can be following truncation and still be retained (*p*) = 0.75 total read length, maximum number of low PHRED scores that can be observed in direct succession before truncating a sequence read (*q*) = 3, maximum number of ambiguous (i.e., N) base calls (*n*) = 0, minimum sequence count (*c*) = 0.005% [12] and sorted into amplicon sequence variants using (ASVs) using a trim length of 200 in Deblur [13]. The resultant QIIME2 abundance and taxonomy artifacts were exported to BIOM format [14] for processing in R v. 3.5.3 [15]. The bacterial abundance data were imported into R using the biomformat package v. 0.4.0^19^ and combined with the taxonomy and sample information using phyloseq v. 1.26.1 [16]. Chloroplasts and mitochondrial contaminants were removed, and abundances standardized to the *Aliivibrio fischeri* spike. Duplicate samples were removed, as were any samples or taxa with zero abundance sums. There were 10,882 unique ASVs with an average of 249 ASVs per sample. ASVs were classified using a 342F/806R-trained V3/V4 SILVA database [17].

#### 2.5.2 Fungi

In total, 113,378,162 forward and reverse raw sequence reads were produced with an average of 26,244 paired reads per sample. Sequences were processed using QIIME2 v2019.7 [11]. First primer sequences were removed. Sequences were quality filtered using the default parameters and arranged as amplicon sequence variants (ASVs) using a forward truncation length of 180 bp and reverse truncation length of 120 bp in DADA2. There were 14,944 unique ASVs with an average of – 43 ASVs per root and 105 ASVs per rhizosphere sample. The average sequence length for both root and rhizosphere was 238 base pairs. ASVs were classified using UNITE database v 8.0 [18]. The QIIME2 abundance and taxonomy artifacts were combined and converted to BIOM format [14] for processing in R v. 3.5.3 [15]. The fungal abundance data were imported into R using the biomformat package v. 0.4.0 and combined with the taxonomy and sample information using phyloseq v. 1.26.1.[16]. Duplicate samples, and samples or taxa with zero abundance sums were removed.

### 2.6 Statistical analysis

Initial exploration of the composition of microbial community was assessed using Principal Component Analysis (PCA) and compositional differences quantified using a Permutational Analysis of Variance (PERMANOVA) in the vegan v. 2.5-6 package in R [19]. First, we removed any taxa with a mean read count ≤ 1 and/or samples with ≤ 1 non-zero value. Next, to address the compositional nature of the canola microbiome dataset Zeros in the dataset were replaced using a Bayes-Laplace approach using the zCompositions v. 1.2.0 R package and then transformed using a centered-log ratio (CLR) transformation using the CoDaSeq v. 0.99.3 package [20], [21], [22]. The CLR mitigates the issue that differences among microbial taxa are not linear by turning the read counts into a ratio abundance (abundance normalized by the geometric mean abundance of all taxa per sample). CLR allows us to retain the relationships among samples and also puts the data in linear (Aitchison) space where we can apply linear statistical techniques such as PCA. The geometric mean centres the abundance values such that the average relative abundance is zero and therefore above average abundances will be positive and below average negative. Alpha diversity was calculated using vegan v. 2.5-6 in R [19].

## Funding

This work is supported by a grant from the Plant Phenotyping and Imaging Research Centre (P2IRC) to BLH, EGL, KS, and SDS. P2IRC is a digital agriculture research center funded by the Canada First Research Excellence Fund (CFREF) from the Natural Sciences and Engineering Research Council (NSERC), managed by the Global Institute for Food Security (GIFS), and located at the University of Saskatchewan (U of S).

## Declaration of Competing Interest

The authors declare no competing interests

## Appendix Supplementary materials

Image, application 1

Image, application 2

## References

[1] Downey R.K., Rimmer S.R. (1993). Agronomic improvement in oil seed. Brassicas Adv. Agron..

[2] Rakow G. (2004). Species origin and economic importance of Brassica. Brassica.

[3] Lay C.Y., Bell T.H., Hamel C., Harker K.N., Ramona M., Greer C.W., Yergeau É., St-Arnaud M. (2018). Canola root–associated microbiomes in the Canadian Prairies. Front. Microbiol..

[4] Floc'h J.B., Hamel C., Harker K.N., St-Arnaud M. (2020). Fungal communities of the canola rhizosphere: keystone species and substantial between-year variation of the rhizosphere microbiome. Microbial Ecology.

[5] Bazghaleh N., Hamel C., Gan Y., Tar'an B., Knight J.D. (2015). Genotype-specific variation in the structure of root fungal communities is related to chickpea plant productivity. Appl. Environ. Microbiol..

[6] Turner T.R., James E.K., Poole P.S. (2013). The plant microbiome. Genome Biol.

[7] Busby P.E., Soman C., Wagner M.R., Friesen M.L., Kremer J., Bennett A., Morsy M., Eisen J.A., Leach J.E., Dangl J.L. (2017). Research priorities for harnessing plant microbiomes in sustainable agriculture. PLoS Biol..

[8] Bazghaleh N., Mamet S.D., Bell J.K., Morales Moreira Z., Taye Z.M., Williams S., Arcand M., Lamb E., Shirtliffe S., Vail S., Siciliano S.D., Helgason B. (2020). "An intensive multilocation temporal dataset of fungal communities in the root and rhizosphere of *Brassica napus*. Harvard Dataverse.

[9] Mori H., Maruyama F., Kato H., Toyoda A., Dozono A., Ohtsubo Y., Kurokawa K. (2014). Design and experimental application of a novel non-degenerate universal primer set that amplifies prokaryotic 16S rRNA genes with a low possibility to amplify eukaryotic rRNA genes. DNA Research.

[10] Toju H., Tanabe A.S., Yamamoto S., Sato H. (2012). High-coverage ITS primers for the DNA-based identification of ascomycetes and basidiomycetes in environmental samples. PloS one.

[11] Bolyen E. (2019). QIIME 2 : Reproducible, interactive, scalable, and extensible microbiome data science. Nat. Biotechnol..

[12] Bokulich N.A., Subramanian S., Faith J.J., Gevers D., Gordon J.I., Knight R., Caporaso J.G. (2013). Quality-filtering vastly improves diversity estimates from Illumina amplicon sequencing. Nat. Methods.

[13] Amir A., Daniel M., Navas-Molina J., Kopylova E., Morton J., Xu Z.Z., Knight R. (2017). Deblur Rapidly Resolves Single-Nucleotide Community Sequence Patterns. Am. Soc. Microbiol..

[14] McDonald D., Clemente J.C., Kuczynski J., Rideout J.R., Stombaugh J., Wendel D., Caporaso J.G. (2012). The Biological Observation Matrix (BIOM) format or: How I learned to stop worrying and love the ome-ome. GigaScience.

[15] R Core Team (2018). R: A language and environment for statistical computing. https://www.R-project.org/.

[16] McMurdie P.J., Holmes S. (2013). Phyloseq: An R Package for Reproducible Interactive Analysis and Graphics of Microbiome Census Data. PLoS ONE.

[17] Quast C., Pruesse E., Yilmaz P., Gerken J., Schweer T., Yarza P., Glöckner F.O. (2013). The SILVA ribosomal RNA gene database project: Improved data processing and web-based tools. Nucleic Acids Res..

[18] UNITE Community (2019). UNITE general FASTA release for Fungi. Version 18.11.2018.

[19] Oksanen J., Kindt R., Legendre P., O'Hara B., Stevens M.H.H, Oksanen M.J., Suggests M.A.S.S (2007). The vegan package. Commun. Ecol. Package.

[20] M. J. Palarea-albaladejo, Package ‘zCompositions’. (2019).

[21] Gloor G.B., Macklaim J.M., Pawlowsky-Glahn V., Egozcue J.J. (2017). Microbiome datasets are compositional: And this is not optional. Front. Microbiol..

[22] Gloor G.B., Reid G. (2016). Compositional analysis: a valid approach to analyze microbiome high-throughput sequencing data. Can. J. Microbiol..

