# Supplementary material for: An intensive multilocation temporal dataset of fungal and bacterial communities in the root and rhizosphere of Brassica napus
Source: Data Brief. 2020 Aug 7;31:106143. doi: 10.1016/j.dib.2020.106143 (PMC7486468; doi:10.1016/j.dib.2020.106143)
Supplement: Supplementary file 2 [file mmc2.docx]

**Fig. S1:** Soil zones of Saskatchewan with study sites indicated in red font.
